# Supplementary material for: Precision improvement for indoor positioning based on fuzzy inference system with ultra-wideband wireless communications
Source: PLoS One. 2026 May 21;21(5):e0349251. doi: 10.1371/journal.pone.0349251 (PMC13193531; doi:10.1371/journal.pone.0349251)
Supplement: Attachment — Submitted filename: Response to reviewers.docx [file pone.0349251.s002.docx]

Journal: PLOS One

Manuscript ID: PONE-D-25-27749

Article: Precision improvement for indoor positioning based on fuzzy inference system with ultra-wideband wireless communications

Authors: Shu-Hung Lee, Shu-Wai Chang, Yung-Fa Huang*, Yung-Hoh Sheu*

Comments and Suggestions for Authors

**Reviewer #1:**

1. Is the AOC method limited to a specific type of NLOS environment or does it work well in different conditions (e.g. with walls, different obstacles)?

**Response:** Thank you for this insightful question. We have added a discussion on this point in Section 3.5 (see revised manuscript).

**Added Text (Section 3.5, in lines 251-261):**

**“**3.5.	Discussion on AOC Robustness in Diverse NLOS Environments

The AOC method demonstrates consistent error reduction across various NLOS conditions encountered in our experimental setup, which included walls, office partitions, and intermittent human obstructions. As shown in Table 2, test points located behind obstacles (e.g., TP_C_, TP_F_, TP_I_) still achieved significant error reduction, confirming the method’s applicability in common indoor NLOS scenarios.

However, the performance of AOC is influenced by the spatial consistency of systematic errors within each zone. In environments where error patterns change rapidly due to highly dynamic or metallic-dense obstacles, the pre-calibrated offset values may require more frequent updates. Future implementations could integrate real-time environmental sensing or adaptive learning mechanisms to maintain accuracy in such challenging conditions.

Nevertheless, for most typical indoor environments—such as offices, warehouses, and smart homes—the AOC method provides a computationally efficient and effective means of mitigating NLOS-induced positioning errors.”

2. Why was fuzzy triple std chosen as the best input membership function?

**Response:** We thank the reviewer for this important methodological question. The choice of the “fuzzy triple standard deviation” (triple std) membership function was not arbitrary but was specifically tailored to address the unique data characteristics observed in our UWB/AoA positioning system under NLOS conditions. In the revised Section 4.2.1, we have added an explanation detailing why the triple std function is superior for our case.

**Added Text (Section 4.1, Paragraph 3, in lines 299-305):**

“The selection of the specific “fuzzy triple standard deviation” (triple std) membership function was driven by the need to effectively model this observed data characteristic—namely, the biased and overlapping distributions caused by systematic NLOS errors. Compared to conventional symmetric membership functions (e.g., standard triangular or Gaussian), the triple std function offers two key advantages for our application: (1) It is inherently data-driven, anchored on the median and scaled by the standard deviation of the training data, which automatically accounts for the distribution's shift and spread. (2) Its asymmetric design, with core (±1.5σ) and support (±3σ) intervals, provides a more flexible and representative fuzzy set for data points that do not conform to idealized, symmetric clusters. This flexibility is crucial for accurately classifying coordinates in the presence of NLOS-induced uncertainty.”

3. Could you elaborate on the number and type of input and output variables of the fuzzy inference system used for area detection?

**Response:** Thank you for requesting this clarification. We have revised the manuscript by adding a new, dedicated overview paragraph at the beginning of Section 4.2.

**Added Text (Section 4.2, in lines 307-312 and 318-323):**

“The proposed system for indoor area recognition is a Mamdani-type fuzzy inference system (FIS) with a two-input, one-output architecture, designed for efficient and interpretable classification of measured coordinates into one of twelve predefined zones (A–L).

4.2.1. Input stage

In this study, the fuzzy inference system (FIS) employs two input variables: $x$ and $y$, representing the measured $x$-coordinate and $y$-coordinate of the target tag, respectively.”

“4.2.2. Output stage

The fuzzy inference system (FIS) in this study has a **single output variable,** $z$**,** which represents the recognized region. This variable corresponds to a total of 12 possible regions, with its linguistic terms denoted as $F_{3}^{l_{3}}\in\left\{ A, B,\ldots, L \right\}, \mathrm{where}l_{3}=1, 2,\ldots, 12.$ Triangular membership functions are used to define these output terms. These functions are preferred for their structural simplicity and parameter efficiency—requiring only the specification of the peak position and the two side slopes to fully describe their shape.”

4. Please provide more details about the layout of the twelve test points used in the experiments. Are these points uniformly distributed in the test area or are they located at specific points with more NLOS challenges?

**Response:** Thank you for this suggestion. We have added a clarification at the end of Section 3.1. The new text reads:

**Added Text (Section 3.1, in lines 186-190):**

“This uniform distribution of test points across the experimental area allows for a comprehensive evaluation of the positioning system. Points at varying distances and angles relative to the base station are included. Specifically, test points located at the far corners of the environment (e.g., **TP_C_**, **TP_F_**, **TP_I_**, **TP_L_**) represent more demanding NLOS scenarios due to extended signal travel distances and increased potential for signal blockage and multipath interference.”

5. Describe the hardware used in UWB and AoA used in the experiments.

**Response:** We appreciate this request for clarity. A new subsection, 2.5 Experimental hardware setup, has been added to the manuscript. It details the core components.

**Added Text (Section 2.5, in lines 172-179):**

“2.5.	Experimental hardware setup

The UWB ranging and communication were implemented using Decawave DW1000 transceiver modules (compliant with IEEE 802.15.4-2011 UWB standard). A custom-designed four-element linear antenna array with an element spacing of 5 cm was used at the base station to enable AoA estimation. The tag node consisted of a single DW1000 module with an omnidirectional antenna. An ESP32 microcontroller served as the processing unit on both the base station and the tag, handling signal timing, data packaging, and communication. The base station communicated with a central processing server via a Wi-Fi module (ESP32 integrated) using WebSocket protocol for real-time data transfer. All devices were mounted on tripods at a fixed height of 1.2 meters to maintain consistency throughout the experiments.”

**Reviewer #2:**

1. Clarify the motivation.

**Response:** Thank you very much for the valuable suggestion to clarify the motivation of our work. The core motivation of this study has been added in the revised manuscript.

**Added Text (Section 1, Paragraph 5, in lines 71-72):**

**“The primary motivation of this work is to address the dual challenge in practical UWB/AoA positioning: mitigating NLOS-induced errors when the target zone is known, and accurately identifying the target zone when it is unknown.**”

2. Highlight the novelty more explicitly.

**Response:** Thank you very much for the valuable suggestion to clarify the novelty of our work. We have now explicitly highlighted our three key contributions in the revised introduction.

**Added Text (Section 1, Paragraph 5, in lines 72-78):**

“To this end, the novelty of this paper is threefold: (1) We propose the Adaptive Offset Cancellation (AOC) method to compensate for systematic positioning errors in known target zones. (2) We introduce a novel fuzzy inference system utilizing a “fuzzy triple standard deviation” input membership function, which is tailored to handle the biased and overlapping coordinate distributions in NLOS settings, for high-accuracy area recognition when the target zone is unknown. (3) We integrate these two components into a unified FAOC (Fuzzy-AOC) framework, which first identifies the target zone and then applies zone-specific error compensation, demonstrating significant overall positioning accuracy improvement in real-world-like scenarios.”

3. Provide a short description of the fuzzy membership function types.

**Response:** We thank the reviewer for this suggestion. In the revised manuscript, we have added a concise description of the membership function types used in both the input and output stages within Sections 4.1 and 4.2.2.

**Added Text (Section 4.1 and 4.2.2, in lines 299-305, and 322-324 ):**

For Input Membership Functions : “The selection of the specific “fuzzy triple standard deviation” (triple std) membership function was driven by the need to effectively model this observed data characteristic—namely, the biased and overlapping distributions caused by systematic NLOS errors. Compared to conventional symmetric membership functions (e.g., standard triangular or Gaussian), the triple std function offers two key advantages for our application: (1) It is inherently data-driven, anchored on the median and scaled by the standard deviation of the training data, which automatically accounts for the distribution's shift and spread. (2) Its asymmetric design, with core (±1.5σ) and support (±3σ) intervals, provides a more flexible and representative fuzzy set for data points that do not conform to idealized, symmetric clusters. This flexibility is crucial for accurately classifying coordinates in the presence of NLOS-induced uncertainty”

For Output Membership Functions : “Triangular membership functions are used to define these output terms. These functions are preferred for their structural simplicity and parameter efficiency—requiring only the specification of the peak position and the two side slopes to fully describe their shape.”

4. Picture Quality of all the Figures should be improved.

**Response:** We acknowledge the reviewer’s concern regarding figure quality and sincerely apologize for the low-resolution figures in the initial submission. All figures (Figs. 1–12) have been re-rendered in high resolution (300 DPI, .tif format) for clarity in the revised manuscript.

5. Flowchart representation should be refined for better understanding.

**Response:** We sincerely appreciate the reviewer’s suggestion regarding the visual representation of our method. We agree that clear flowcharts are essential for comprehending the system architecture. Accordingly, we have completely redrawn both the Training Procedure (Fig 4) and the Testing Procedure (Fig 7) to enhance their logical flow and readability.

“

**Fig 4. Proposed training procedure for parameter estimation.**

**Fig 7. Testing procedure for fuzzy area recognition.”**

6. Include the recent findings & survey (2024 & 2025).

**Response:** We gratefully acknowledge the reviewer's suggestion to update the literature review. We have revised the Introduction section to incorporate state-of-the-art findings from 2024 and 2025. Specifically, we added citations [31, 32, 33] to provide a comprehensive overview of recent UWB positioning trends. Furthermore, we referenced significant 2025 studies [34, 35] that utilize fuzzy logic-enhanced approaches for NLOS mitigation. These recent works strongly support our motivation for using a Fuzzy Inference System (FIS), demonstrating its continued relevance and effectiveness in handling complex indoor positioning challenges.

**Added Text (Section 1, Paragraph 4 , in lines 61-66):**

“Recent advancements in the field have further highlighted the critical need for robust NLOS mitigation strategies. Comprehensive surveys [31, 32] indicate that while UWB remains a dominant technology for high-precision indoor localization, current research trends are shifting towards integrating adaptive algorithms to handle environmental uncertainties [33]. Notably, contemporary studies have successfully demonstrated the efficacy of hybrid approaches that incorporate fuzzy logic for NLOS identification and mitigation, proving that fuzzy-based inference remains a powerful tool for enhancing robustness in complex multipath environments [34, 35].”

7. Mathematical notations to be included properly.

**Response:** We sincerely thank the reviewer for the suggestion regarding the rigor of mathematical notations. We have conducted a thorough review and revision of all mathematical expressions throughout the manuscript to ensure they adhere to formal academic standards.

**Modified Text (Equations 3-6, and 9, in lines 141-150, and 328-331):**

“The calculation method for the average offset of the *x* and *y* coordinates of the *i*-th training test point can be expressed by

| $X_{O,i}$ = $\bar{x}_{train,i}-T_{x,i},$ | (3) |
| --- | --- |

and

| $Y_{O,i}$ = $\bar{y}_{train,i}-T_{y,i},$ | (4) |
| --- | --- |

respectively, where $\bar{x}_{train,i}$and $\bar{y}_{train,i}$ represent the average coordinates after applying moving average filtering to the training data, and $T_{x,i}$ and $T_{y,i}$are the true coordinates of the test point for x and y, respectively.

After obtaining the offsets of x and y in each region, the experiment first processes the measured signals using moving average filtering, and then corrects them based on the offset of the region where the test point is located. The corrected coordinates of the test point ($\acute{x}_{i}$, $\acute{y}_{i}$) are

| $\acute{x}_{i}$ = $\bar{x}_{i}$ $-$ $X_{O,i},$ | (5) |
| --- | --- |
| $\acute{y}_{i}$ = $\bar{y}_{i}$ $-$ $Y_{O,i},$ | (6) |

where $X_{O,i}$ and $Y_{O,i}$ are the offsets of the x and y coordinates for each test point, where *i* =1~12, and $\bar{x}_{i}$ and $\bar{y}_{i}$ are the average coordinates after applying moving average filtering to the *i*-th test point.”

“

The fuzzy inference engine operates on a set of linguistic IF-THEN rules. To formalize the inference logic, the *k*-th fuzzy rule *R*_k_ is represented as follows:

$R_{k}:\mathrm{IF} x is F_{1}^{l_{1}} \mathrm{AND} y is F_{2}^{l_{2}} \mathrm{THEN} z= F_{3}^{l_{3}}$,

(9)

where $F_{1}^{l_{1}}$, $F_{2}^{l_{2}}$and $F_{3}^{l_{3}}$are the linguistic terms of the input variables *x*, *y* and the output variable *z*, respectively, and *l*_1_=1, 2, 3, *l*_2_=1,2, .., 4, *l*_3_ and the index of rule *k* = 1, 2, …, 12.”

8. Briefly indicate why the procedure is divided into two phases.

**Response:** Thank you very much for this comment. We have clarified this rationale in the revised Section 3.2.

**Added Text (Section 3.2, in lines 199-202):**

“The experimental procedure is intentionally structured into two distinct phases—training and testing—to emulate a practical deployment paradigm: an initial long-term calibration period followed by operational use. This separation ensures that the system's error compensation is learned from comprehensive calibration data, preventing overfitting and providing a realistic assessment of performance in a scenario akin to real-world application.”

9. Clarify the role of the moving average filter in both phases.

**Response:** Thank you very much. The distinct roles are now explicitly described in Section 3.2. In the training phase, it is used to suppress high-frequency noise and yield a stable, representative average position for calculating offsets. In the testing phase, its role is to provide a smoothed, real-time coordinate estimate by mitigating instantaneous measurement noise, thus preparing a cleaner signal for the subsequent correction step.

**Added Text (Section 3.2, in lines 208-210, and 218-220):**

training phase “to suppress high-frequency noise and yield a stable, representative average position **(**$\bar{x}_{train,i} ,$ $\bar{y}_{train,i})$ for each point”

testing phase “to provide a smoothed, real-time coordinate estimate by mitigating instantaneous measurement noise, thus preparing a cleaner signal for the subsequent correction step.”

10. Mention sampling duration or amount of data collected.

**Response:** Thank you very much for this comment. We have added specific details in Section 3.2 in the revised manuscript.

**Added Text (Section 3.2, in lines 203-205, and 215-216):**

For the training phase: *”*Data collection spanned seven days to capture a wide range of environmental variations. At each of the 12 test points, TP_A_ to TP_L_, 7,000 consecutive positioning samples were collected at a sampling rate of 1 Hz.”

For the testing phase: “A comparable volume of test samples, 7,000 samples per point at 1 Hz, was collected to match the training data's statistical scale.”

11. State how the offset is stored and associated with specific zones.

**Response:** Thank you for this suggestion. This is clearly stated in Section 3.2. in the revised manuscript.

**Added Text (Section 3.2, in lines 212-213, and 223-224):**

After calculation, *“*These 12 offset pairs, each uniquely associated with a zone label (A through L), were stored in a lookup table.*”*

During testing, *“*the offset pair corresponding to the identified (or known) zone is fetched from the lookup table and subtracted from the smoothed coordinates.”

12. Strengthen the last sentence by clearly stating the outcome.

**Response:** Thank you for this suggestion. We have revised the concluding paragraph to end with a stronger, more declarative statement of the main achievement.

**Added Text (Section 4.3, Last Paragraph, in lines 365-368):**

“Overall, the proposed FAOC method—which sequentially identifies the target area via the fuzzy inference system before applying AOC—successfully reduces the average positioning error across all twelve regions from 69.03 cm to 44.48 cm, achieving a significant improvement rate of 40.9%.”

13. Add an overall summary statement in the conclusion.

**Response:** Thank you for this suggestion. We agree that a high-level summary strengthens the conclusion. We have added an opening sentence to Section 5.

**Added Text (Section 5, in lines 370-371):**

“This study presents a comprehensive framework for enhancing UWB/AoA-based indoor positioning accuracy in NLOS environments by addressing both error compensation and area recognition.”

14. Add a future work statement.

**Response:** We appreciate this constructive suggestion. A future work statement has been added in Section 5 in the revised manuscript.

**Added Text (Section 5, in lines 376-380):**

“Building on these promising results, future research will focus on several key areas. These include developing online adaptive offset update mechanisms for highly dynamic environments, integrating fuzzy logic with deep learning for more robust NLOS identification, and deploying the FAOC framework on low-power embedded platforms to facilitate practical applications in smart warehouses and asset tracking.”
