# Supplementary material for: Precision improvement for indoor positioning based on fuzzy inference system with ultra-wideband wireless communications
Source: PLoS One. 2026 May 21;21(5):e0349251. doi: 10.1371/journal.pone.0349251 (PMC13193531; doi:10.1371/journal.pone.0349251)
Supplement: Attachment — Submitted filename: Response_to_Reviewers_auresp_2.docx [file pone.0349251.s003.docx]

Dear Editor and Reviewers,

We would like to express our sincere gratitude for the time and effort you have dedicated to reviewing our manuscript (Manuscript ID: PONE-D-25-27749) entitled “Precision improvement for indoor positioning based on fuzzy inference system with ultra-wideband wireless communications.” We greatly appreciate the constructive comments and valuable suggestions provided by the reviewers, which have helped us substantially improve the quality and clarity of our work.

We have carefully addressed all the comments raised by the reviewers. Below, we provide a point-by-point response to each comment, along with a description of the corresponding modifications made to the manuscript. All changes have been highlighted in the revised manuscript.

**Response to Reviewer #1:**

1. Given that your work focuses on improving accuracy in NLOS environments, how can your system adapt to environmental changes in real time?

**Response:** We thank the reviewer for raising this important point regarding real-time adaptability. In the current implementation, the Adaptive Offset Cancellation (AOC) method relies on pre-calibrated zone-specific offsets obtained during an initial training phase. While this approach effectively mitigates systematic errors in static or slowly varying environments, we acknowledge its limitation in highly dynamic scenarios. To address this concern, we have added a discussion in the Conclusions section outlining our future work on an online adaptive offset update mechanism, which will enable the system to adapt to instantaneous environmental changes without manual recalibration.

**Added Text (Section 5, in lines 392-399):**

“Building on these promising results, our future research will first address the challenges of real-time environmental dynamics and long-term stability. We recognize that the current AOC method, which relies on pre-calibrated offsets, may have limitations in highly dynamic scenarios. Therefore, we plan to develop an online adaptive offset update mechanism. This mechanism will continuously monitor incoming positioning data and dynamically refine zone-specific offset values using sliding window estimation or recursive learning techniques. By doing so, the system will be capable of adapting to instantaneous environmental changes—such as moving obstacles or varying multipath conditions—while maintaining accuracy over extended periods without requiring manual recalibration. This advancement will transform the current FAOC framework into a truly adaptive real-time positioning system.”

2. In the experiments, your test environment is limited to a 600x800 cm space. How do you think the FAOC method will scale in larger or more complex environments (such as multi-story buildings or smart cities)?

**Response:** We appreciate the reviewer's concern regarding scalability. While our current experiments were conducted in a controlled indoor environment, the proposed FAOC method is designed to be scalable and modular. In larger or more complex environments, the system can be extended by increasing the number of zones and corresponding fuzzy rules. For multi-story buildings, a hierarchical fuzzy system could be implemented to first identify the floor and then the zone. We have added a discussion on scalability in the revised manuscript.

**Added Text (Section 4.4, in lines 375-383):**

“4.4 Scalability and Applicability in Complex Environments

While the experiments in this study were conducted in a controlled indoor environment measuring 600 cm × 800 cm, the proposed FAOC method is designed with scalability in mind. In larger or more complex environments, such as multi-story buildings or smart city infrastructures, the system can be extended by increasing the number of zones and corresponding fuzzy rules. The modular structure of the fuzzy inference system allows for easy reconfiguration by adjusting membership functions and rule bases. For multi-story scenarios, a hierarchical fuzzy system could be implemented to first identify the floor level and then the specific zone within that floor. Furthermore, the computational efficiency of the FAOC method makes it suitable for distributed deployment across multiple base stations, enabling seamless coverage expansion. These scalability features position the FAOC method as a viable solution for a wide range of indoor positioning applications.”

3. You have compared your fuzzy inference system with clear area recognition and K-means methods. The question arises as to how your method performs compared to more advanced methods such as machine learning and big data-based models for reducing NLOS errors?

Response: We agree that comparison with more advanced methods is important. In this study, we focused on interpretable and computationally lightweight fuzzy systems suitable for real-time embedded applications. However, we recognize the growing potential of machine learning and deep learning approaches for NLOS mitigation. In our ongoing work, we are investigating hybrid models that combine fuzzy logic with neural networks (e.g., ANFIS) and deep learning classifiers. We have included this direction in the future work section.

**Added Text (Section 5, in lines 400-403):**

“In parallel, we will pursue several avenues to further enhance positioning accuracy towards sub-20 cm levels across all zones. First, we plan to integrate fuzzy logic with advanced machine learning techniques, such as deep neural networks and hybrid neuro-fuzzy systems (e.g., ANFIS), to improve NLOS identification and mitigation. Comparative studies with state-of-the-art machine learning and big data-based localization models will also be conducted to validate and refine our approach.”

4. In the results, you have shown that AOC was able to reduce the positioning error by an average of 29.38 cm, but in some cases the final error is still around 20 cm. What plans do you have to reduce this error in future versions? Can improvements in signal processing or hardware help to achieve sub-20 cm accuracy?

**Response:** We thank the reviewer for this constructive comment. Achieving sub-20 cm accuracy consistently across all zones is indeed a goal of our ongoing research. Future improvements may include enhanced signal processing techniques, hardware upgrades, and multi-sensor fusion approaches. We have elaborated on these potential enhancements in the revised manuscript.

**Added Text (Section 5, in lines 404-407):**

“Second, from a hardware and signal processing perspective, we will explore the integration of higher-precision UWB modules, improved antenna arrays for more accurate AoA estimation, and advanced filtering techniques such as adaptive Kalman filtering. Finally, multi-sensor fusion approaches that combine UWB with inertial measurement units (IMU) or visual sensors will be investigated to complement UWB measurements in particularly challenging NLOS conditions.”

5. Is your method for correcting offset values stable enough over time and with changing environmental conditions?

**Response:** We appreciate the reviewer's insightful question regarding long-term stability. In our current experimental setup, the training phase spanned seven days to capture typical daily variations, and the pre-calibrated offsets demonstrated effective error reduction during the subsequent testing phase. However, in environments with significant long-term changes (e.g., furniture rearrangement, new obstacles), the pre-calibrated offsets may degrade. To ensure sustained performance, our future work will focus on implementing the same online adaptive offset update mechanism described in response to Comment 1. This approach will maintain the system's stability and accuracy over extended periods, even as environmental conditions evolve.

**Added Text (Section 5, in lines 396-398):**

“...adapting to instantaneous environmental changes...while maintaining accuracy over extended periods without requiring manual recalibration.”

6. Is this system currently compatible with systems on the market that use technologies such as Wi-Fi or UWB?

**Response:** Yes, our system is designed to be compatible with commercial UWB hardware, specifically the Decawave DW1000 modules, which are widely used in industry. The proposed FAOC method operates at the software level and can be integrated into existing UWB-based positioning systems without hardware modification. Furthermore, the fuzzy inference engine can be implemented on low-cost microcontrollers, making it suitable for embedded deployment. We have clarified this compatibility aspect in the revised manuscript.

**Added Text (Section 1, in lines 78-81, Section 2.5, in lines 182-185, and Section 5, in lines 408-412):**

“Moreover, the proposed FAOC framework is designed to be compatible with existing commercial UWB hardware and can be integrated into current indoor positioning systems with minimal software adaptation, facilitating its practical deployment in real-world applications.”

“It is worth noting that the proposed FAOC method is implemented at the software level and is fully compatible with commercially available UWB systems, such as those based on Decawave DW1000 modules. The fuzzy inference engine can be executed on low-cost microcontrollers (e.g., ESP32), making it suitable for embedded deployment without requiring hardware modifications.”

“Finally, to facilitate practical deployment, we will focus on scaling the FAOC framework to larger and more complex environments, such as multi-story buildings, through hierarchical fuzzy systems and distributed base station architectures. Concurrently, we aim to deploy the system on low-power embedded platforms (e.g., ARM Cortex-M series) to validate its compatibility with commercial UWB hardware and its suitability for real-world applications like smart warehouses and asset tracking.”

We believe that the revisions have significantly strengthened the manuscript and have addressed all the concerns raised by the reviewer. We thank the Editor and Reviewer again for their time and valuable feedback. We look forward to your further consideration.

Sincerely,

Yung-Fa Huang

Ph.D. Distinguished Professor

Department of Information and Communication Engineering Chaoyang University of Technology

Taichung, Taiwan

Email: yfahuang@cyut.edu.tw
